# Cerebrospinal Fluid Neopterin Analysis in Neuropediatric Patients: Establishment of a New Cut Off-Value for the Identification of Inflammatory-Immune Mediated Processes

**DOI:** 10.1371/journal.pone.0083237

**Published:** 2013-12-18

**Authors:** Marta Molero-Luis, Sergio Fernández-Ureña, Iolanda Jordán, Mercedes Serrano, Aida Ormazábal, Àngels Garcia-Cazorla, Rafael Artuch, the Neopterin working group

**Affiliations:** 1 Clinical Biochemistry and Neuropediatrc Departments, University Hospital Sant Joan de Déu, and Centre for Biomedical Research on Rare Diseases (CIBER-ER), Instituto de Salud Carlos III, Barcelona, Spain; 2 Intensive Pediatric Care Unit Service, University Hospital Saint Joan de Déu, Barcelona, Spain; 3 Molecular Microbiology Department, Microbiology Service, University Hospital Sant Joan de Déu, Barcelona, Spain; University of Sydney, Australia

## Abstract

**Objective:**

A high level of cerebrospinal fluid (CSF) neopterin is a marker of central nervous system inflammatory-immune mediated processes. We aimed to assess data from 606 neuropediatric patients, describing the clinical and biochemical features of those neurological disorders presenting CSF neopterin values above a new cut-off value that was defined in our laboratory.

**Methods:**

To establish the new CSF neopterin cut-off value, we studied two groups of patients: Group 1 comprised 68 patients with meningoencephalitis, and Group 2 comprised 52 children with a confirmed peripheral infection and no central nervous system involvement. We studied 606 CSF samples from neuropediatric patients who were classified into 3 groups: genetic diagnosis (A), acquired/unknown etiologic neurologic diseases (B) and inflammatory-immune mediated processes (C).

**Results:**

The CSF neopterin cut-off value was 61 nmol/L. Out of 606 cases, 56 presented a CSF neopterin level above this value. Group C had significantly higher CSF neopterin, protein and leukocyte values than the other groups. Sixteen of twenty-three patients in this group had a CSF neopterin level above the cut-off, whereas three and seven patients presented increased leukocyte and protein values, respectively. A significant association was found among CSF neopterin, proteins and leukocytes in the 606 patients. White matter disturbances were associated with high CSF neopterin concentrations.

**Conclusions:**

Although children with inflammatory-immune mediated processes presented higher CSF neopterin values, patients with other neurological disorders also showed increased CSF neopterin concentrations. These results stress the importance of CSF neopterin analysis for the identification of inflammatory-immune mediated processes.

## Introduction

Neopterin is a pterin containing a 2-amino-4-oxo-pyrazine-pyrimide (pterin) ring and is formed from guanosine-triphosphate in the synthetic pathway of tetrahydrobiopterin (BH_4_) [1]. BH_4_ acts as a cofactor in the rate-limiting enzymatic step of dopamine and serotonin biosynthesis (hydroxylation of tyrosine and tryptophan) [2]. It also acts as a cofactor in the hydroxylation of phenylalanine to tyrosine [3] and plays a role in the inducible nitric oxide synthase reaction [2]. Moreover, neopterin is a direct response to T-helper cell 1 stimulation by interferon-γ [4], [5], the central cytokine involved in the activation of the cellular immune system. Therefore, neopterin is a sensitive indicator in immune-mediated and inflammatory disorders [1], [6]. Its concentration in biological fluids may be useful for the diagnosis of inflammatory and immune-mediated diseases in which T-helper cell 1 and macrophages are involved [2], [6], [7]. It is also useful for the diagnosis of several genetic conditions affecting BH_4_ biosynthesis and neurotransmitter pathways [8], [9].

Age-matched reference values for CSF neopterin in the pediatric age have scarcely been reported [2], [10], [11], [12] and have been directed towards detecting inborn errors of BH_4_ metabolism. Increased CSF neopterin concentrations have been described in children with bacterial meningitis [13], viral encephalitis, febrile convulsions, cryopyrin-associated periodic syndrome [14], Aicardi-Goutières syndrome [12], opsoclonus-myoclonus [15] and infantile epileptic encephalopathies [16]. Therefore, assessing the CSF neopterin values in a cohort of patients with central nervous system (CNS) inflammatory or immune-mediated diseases seems important as a first step to establish new cut-off values that may indicate when a patient may present an inflammatory-immune event. Furthermore, many pediatric neurological patients end up having a syndromic diagnosis of unknown etiology after extensive metabolic studies [17]. In some of these patients, the presence of high CSF neopterin values might indicate inflammatory-immune mediated processes in the CNS, facilitating the differential diagnosis of other neurometabolic and neurogenetic conditions.

We aimed to establish a new CSF neopterin cut-off value using a cohort of patients with CNS immune-mediated diseases. After defining this new value, we assessed the data from a cohort of 606 neuropediatric patients and described the clinical and biochemical features of those neurological disorders presenting high CSF neopterin values.

## Materials and Methods

### Ethics Statement

All samples from the patients were obtained in accordance with the 2000 revised Helsinki Declaration of 1975. The study was approved by the local ethics committee review board, named “Comitè Ètica Investigació Clínica” from Sant Joan de Déu Foundation. All parents or guardians on behalf of the participants provided the written informed consent to participate in this study.

### Material and methods

We previously established reference values for CSF neopterin concentrations in a cohort of 116 pediatric patients in whom viral or bacterial meningitis, encephalitis and other neurological conditions of non-metabolic origin were ruled out [11]. In that study, no differences were observed in patients older than one month, whose upper limit reference interval for neopterin was established as 34 nmol/L, very similar to that previously reported [6], [10]. To establish the new CSF neopterin cut-off value, we studied two groups of patients, all of them older than one month of age. Group 1 comprised 68 patients (45 males, 23 females, mean age 6.5 years, standard deviation [SD] 5 years, median age 5.2 years, range 1 month–19 years, Table 1) with viral (n = 54) and bacterial meningoencephalitis (n = 14). Confirmed microbiological diagnoses were achieved by specific polymerase chain reaction and bacterial culture methods according to routine standardized procedures. Among the viral meningoencephalitis patients, the main agents were Herpesviruses (n = 4) and Enteroviruses (n = 24). The remaining patients in this subgroup (26/54) had lymphocytic meningoencephalitis under investigation. Regarding bacterial infections, *Streptococcus pneumoniae* (n = 8) and *Neisseria meningitides* (n = 6) were responsible for the disease. White blood cell (WBC), glucose and protein levels in the CSF were analyzed for each sample (all cases showed WBC counts higher than 5 cells/mm^3^). We excluded those samples with elevated WBC counts attributable to contamination with blood after a corrective formula was applied [18]. Group 2 comprised 52 children (40 males, 12 females, mean age 8 months; SD 12 months; median age 5.4 months, age range: 1 month–6 years, Table 1) with confirmed viral (n = 15) or bacterial (n = 37) peripheral infections without CNS involvement.

**Table 1:** CSF biochemical values from subjects with CNS infections and peripheral infections.

Medical Diagnosis	N	CSF Neopterin (nmol/L) median (range)	CSF proteins (mg/mL) median (range)	CSF leukocytes (WBC/mm^3^) median (range)	CSF glucose (nmol/L) median (range)
Viral meningitis	54	159.5 (46–844)	37 (15–499)	92.5 (9–2000)	3.24 (2.48–4.73)
Bacterial meningitis	14	265 (18–1368)	126.5 (14–490)	242.5 (10–18380)	2.14 (0.28–3.58)
**Total Group 1** (Interval age 1 mo–19 y)	68	167 (18–1368)*	40.5 (14–499)*	100 (9–18320)*	3.21 (0.28–4.73)*
Viral peripheral infection	15	45 (23–193)	30 (4–61)	0 (0–15)	3.21 (2.7–4.18)
Bacterial peripheral infection	37	28 (8–282)	19 (10–99)	0 (0–40)	3.57 (2.37–6.33)
**Total Group 2** (Interval age 1 mo–6 y)	52	34.5 (8–282)*	21 (10–99)*	0 (0–40)*	3.41 (2.37–6.33)*

*Significant values were observed in Total Group 1 vs Total Group 2 (U-Mann Whitney test). The data for the CSF variables are shown as median and range in brackets. CSF: cerebrospinal fluid, WBC: white blood cells.

### Neurologic cohort of patients

Between January 2001 and April 2012, 852 CSF samples were analyzed in the Sant Joan de Déu Hospital Laboratory. The samples were collected from patients afflicted by several neurological disorders for which a diagnostic lumbar puncture was indicated. Of the initial group of samples, 242 were excluded for one of the following reasons: traumatic (hematic) puncture and inadequate conditions for sample collection and preservation, such as the lack of rostral-caudal gradient sampling, inappropriate temperature or lack of light protection. Finally, 606 samples (mean age: 4.5 years; SD 4.7 years; age range: 0.16–18 years; sex: 52.6% males) were included in the study and were classified into three different clinical groups: A) patients with confirmed genetic diseases (n = 160), including inborn errors of metabolism of simple and complex molecules, mitochondrial diseases and neurogenetic disorders; B) patients with acquired neurologic disorders (n = 423), including ischemic and hemorrhagic injuries, neurological disorders with motor disturbances, mental retardation plus other neurological signs and psychiatric conditions, leukodystrophy and malformations, epilepsy and epileptic encephalopathies; and C) patients with inflammatory or immune-mediated diseases (n = 23), including Aicardi-Goutières syndrome, acute disseminated encephalomyelitis, Guillain Barré syndrome, anti-NMDA (N-methyl D-aspartate) receptor encephalitis, chronic infantile neurological, cutaneous and articular syndrome, histiocytosis, myasthenia gravis syndrome and brain parasitosis. Detailed information about the diagnoses of the entire cohort is available upon request. Their main clinical and CSF biochemical data are presented in Tables 2 and 3.

**Table 2:** Patients with CSF neopterin levels above 61/l (n = 56).

Clinical groups (patients NP>61 nM/total patients group)	Diagnosis	CSF NP (nmol/l)	CSF proteins (mg/dl)	CSF leukocytes (WBC/mm^3^)	Patients with altered proteins	Patients with altered leucocytes
A. Genetic diseases (n = 10/160, 6.2%)	8 mitochondrial dis., 1 ARCI syndrome, 1 VLCAD def.	68.7 (62.6–153)	26 (16–113)	0 (0–5)	2/10	0/10
B. Acquired neurologic disorders without inflammatory suspicion (n = 30/423, 7.1%)	12 epilepsies, 2 epileptic encephalopathy, 9 white matter dist., 4 motor dist., 2 strokes, 2 necrosis, 1 mental disorder.	97 (61–486)	22 (11–459)	0 (0–70)	4/30	1/30
C. Inflammatory/immune-mediated diseases (n = 16/23, 69.6%)	9 AGS, 3 encephalitis, 1 Anti-NMDA enceph., 1 CINCA sd, 1 brain parasitosis, 1 histiocytosis	238 (67–3010)*	33 (16–447)*	(0–30)*	7/16	3/16
Total patients (n = 56/606)					13/56	4/56

*Inflammatory/immune-mediated group had significantly elevated CSF neopterin (X^2^ = 35.01, p<0.001), CSF proteins (X^2^ = 12.71, p = 0.002) and CSF leukocytes (X^2^ = 6.23, p = 0.044) compared with the other groups. Moreover, up to 69.6% of the patients more frequently had CSF NP>61 nmol/L. Out of 56 patients with a CSF neopterin level above 61 nmol/l, 13 patients had altered CSF proteins and four had altered CSF leucocytes.

AGS: Aicardi-Goutières syndrome; ARCI: arthrogryposis, renal tubular dysfunction, cholestasis, ichthyosis syndrome; anti-NMDA encephalitis: anti-(N-methyl D-aspartate) receptor encephalitis; CINCA: chronic infantile neurological, cutaneous and articular syndrome; VLCAD: very long-chain acyl-CoA dehydrogenase; CSF NP: cerebrospinal fluid neopterin; WBC: white blood cells. Dist: disturbances. The results are shown as the median and interval in brackets.

**Table 3:** Patients with CSF neopterin levels below 61/l (n = 550).

Clinical groups (patients NP>61 nM/total patients group)	CSF NP (nmol/l)	CSF proteins (mg/dl)	CSF leukocytes (WBC/mm^3^)	Patients with altered proteins	Patients with altered leucocytes
A. Genetic diseases (n = 150/161, 92.9%)	14.05 (4.1–60)	20 (4–305)	0 (0–10)	18/150	5/150
Neurologic disorders without inflammatory suspicion (n = 393/422, 93.8%)	14 (3.10–57.5)	21 (1–226)	0 (0–169)	47/393	3/393
Inflammatory/immune-mediated disease (n = 7/23, 30.4%)	15 (11.80–36.3)	39 (15–170)	0 (0–75)	3/7	1/7
Total patients (n = 550/606)				68/550	9/550

Out of 550 patients with CSF neopterin levels below 61 nmol/l, 68 patients had altered CSF proteins, and nine had altered CSF leucocytes. CSF NP: cerebrospinal fluid neopterin. The results are shown as the median and interval in brackets.

### Laboratory studies

In the 606 patients, screening laboratory tests for the diagnosis of metabolic and genetic diseases (lactate, ammonium, amino acids, organic acids, acylcarnitines, glycosaminoglycans, sialotransferrin, fatty acids and karyotype) were performed when indicated, using standard procedures. CSF samples were collected by lumbar puncture as previously reported [11].Neopterin and biopterin determinations were performed by fully oxidation of all isophorms of pterins by MnO_2_. Both total pterins were analyzed by reverse phase high performance liquid chromatography with fluorescence detection following a previously reported procedure [11]. Briefly, the mobile phase consisted of 1 µmol/l potassium phosphate plus methanol (95/5 v/v). Excitation was 350 nm, and emission was 450 nm. Glucose and proteins were analyzed with standard automated spectrometric procedures. WBCs were counted in a manual counting chamber using undiluted CSF. The differential analysis of WBCs was only performed upon increased CSF WBC counts, and the cells were classified into polymorphonuclear and mononuclear leukocytes. The reference values were 70–109 mg/dL (3.9–6.1 nmol/l) for CSF glucose, below 40 mg/dl for proteins and below 5 cells/mm^3^ for WBCs.

### Statistics

All analyses were performed using the SPSS 19.0 software. To establish the new CSF neopterin cut-off, COR curve analysis was applied. The Kolomogorov-Smirnow test was applied to assess the data distribution. Because the data did not follow a Gaussian distribution, different non-parametric tests were applied. The Mann-Whitney U test was used to test for the statistical significance of different CSF variables in Groups 1 and 2. In the entire sample (n = 606), the Spearman correlation test was applied to search for correlations between the CSF neopterin level and the other variables studied in the CSF (biopterin, proteins, leucocytes, glucose) and age. To compare the CSF neopterin level with the other CSF variables among the different clinical groups, the Kruskal-Wallis test was applied. To describe whether high CSF neopterin values were associated with a significantly higher risk of having altered CSF proteins (>40 mg/dl), altered CSF leucocytes (>5 cells/mm^3^), white matter disturbances or the presence of epilepsy, we applied the Chi-square test and computed the Odds ratio for each group. Statistical significance was defined as p<0.05.

## Results

**Figure 1:**
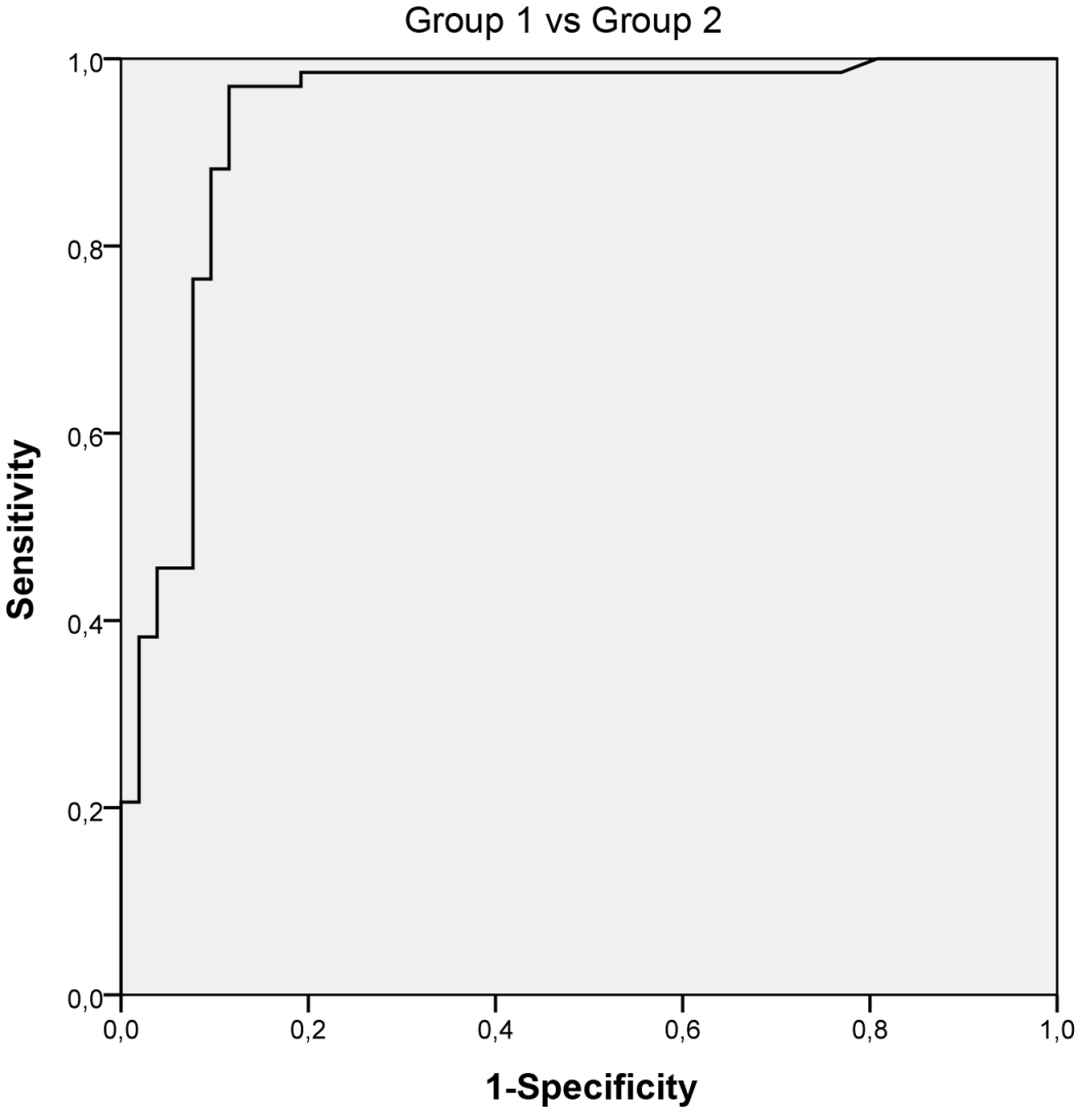
ROC curve to determine the new CSF neopterin cut-off. The samples were classified into two groups according to the condition that they presented either bacterial/viral central infection (n = 68) or peripheral infection (n = 52). The area under the curve was 0.934 (range: 0.883–0.985) with 61 nmol/l as the new cut-off. The sensitivity was 91.3%, and the specificity was 88.5%.

To establish the CSF neopterin cut-off value, a ROC curve between groups 1 (central infections) and 2 (peripheral infections) was created. The best discrimination was provided by the CSF neopterin value of 61 nmol/L (AUC = 0.934, range: 0.883–0.985) with a sensitivity of 91.3% and specificity of 88.5% (Figure 1). Table 1 shows the results of the different CSF laboratory variables analyzed in groups 1 and 2. Significant differences were observed between groups 1 and 2 for all CSF variables (Mann-Whitney U test, p<0.001 for CSF WBC, p<0.001 for CSF neopterin, p = 0.038 for CSF glucose and p<0.001 for CSF proteins).

Neurologic cohort of patients: In the 606 patients, a significant positive correlation was observed for CSF neopterin with CSF proteins (r = 0.178, p<0.001) and leukocytes (r = 0.148, p<0.001) but not with CSF glucose, CSF biopterin and patient age. Given this last finding, a unique age group of patients was considered for subsequent analysis.

**Figure 2:**
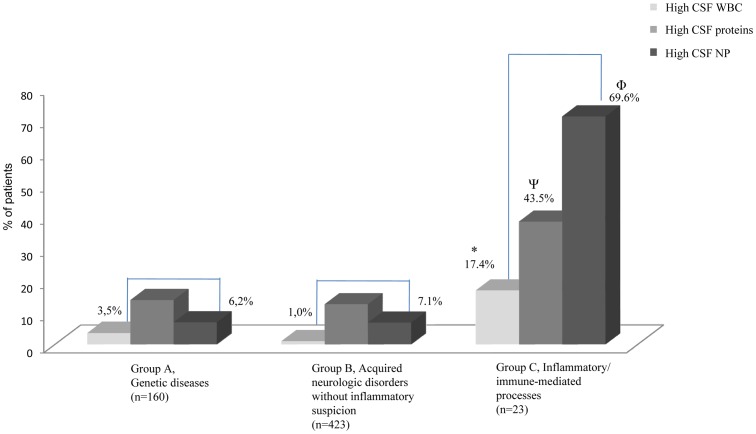
Representation of the 606 neurological patients in the clinical groups. Each group shows the percentage of patients with high CSF WBC, high CSF proteins and high CSF neopterin. The inflammatory/immune-mediated group had a significantly elevated percentage of high CSF WBC (^*^X^2^ = 27.91, p<0.001), high CSF proteins (^Ψ^X^2^ = 17.51, p<0.001) and high CSF NP (^Φ^X^2^ = 103.73, p<0.001) compared with the other groups.

Figure 2 shows the distribution of the 606 neurological patients according to three clinical groups and the percentages of patients with high CSF WBC (>5 cells/mm^3^), high CSF proteins (>40 mg/dL) and high CSF neopterin concentrations (>61 nmol/L). In all groups, the proportion of patients with high CSF neopterin levels was higher than that of high CSF WBC. Of these 606 patients, 56 presented with increased CSF neopterin values according to the 61 nmol/L cut-off value (Table 2). The inflammatory-immune mediated group (n = 23) had significantly higher CSF neopterin values (X^2^ = 35.01, p<0.001), CSF proteins (X^2^ = 12.71, p = 0.002) and CSF leukocytes (X^2^ = 6.23, p = 0.044) compared with the other groups. Moreover, in this group, up to 69.6% of the patients (16 out of 23) had CSF neopterin >61 nmol/l. In contrast, among these 16 patients, three had increased WBC, and seven presented increased proteins (Table 2). Considering the seven patients with CSF neopterin values below 61 nmol/L, one presented increased CSF WBC, and three had increased CSF proteins (Table 3). The etiological diagnoses of these 23 patients are presented in Tables 2 and 3. Remarkably, groups A and B showed a relatively high percentage of patients with increased CSF neopterin levels, with some overlapping with those values found in the group of inflammatory-immune mediated processes (group C, Table 2). Table 3 presents the clinical diagnoses and biochemical findings of the 550 patients with CSF neopterin <61 nmol/l. The proportion of patients with high CSF proteins and high CSF WBC was 68/550 and 9/550, respectively. No differences were observed among the three clinical groups for the different CSF biochemical parameters.

Finally, we explored whether increased CSF neopterin (>61 nmol/L) could be related to a significantly higher risk of having high CSF proteins (>40 mg/dl), high CSF WBC (>5 cells/mm^3^), white matter disturbances or the presentation of epilepsy. The odds ratio showed a significant association between high CSF neopterin and high CSF proteins in the entire patient cohort (1.95-fold higher, CI 95% = 1.1–3.48, p = 0.026) and increased WBC count (3.39-fold higher, CI 95% = 1.44–7.98, p = 0.028). The risk of having alterations in white matter with CSF neopterin >61 nmol/L was only observed in group B (2.30-fold higher, CI 95% = 1.72–3.07, p<0.001). Conversely no increased risk of having epilepsy with CSF neopterin above 61 nmol/l was observed in any group. Additionally, there was no significant risk of having high CSF proteins, high CSF leukocytes, white matter disturbances or epilepsy when the CSF neopterin level was below 61 nmol/L.

## Discussion

Neopterin is released in the first step of the BH_4_ biosynthetic pathway, which is controlled by the GTP cyclohydrolase I enzyme [1]. Some inflammatory cytokines, mainly interferon-γ, selectively stimulate this enzyme in macrophages, leading to the accumulation and excretion of high levels of neopterin in the body fluids of patients with inflammatory or immune-mediated processes [12], [19]. It is known that there is an intrathecal synthesis of neopterin by microglia, which constitutes the innate immune cells of the CNS [2], [20]. As the blood-brain barrier has a low permeability for peripheral neopterin [2], the study of CSF neopterin may be a useful tool for the diagnosis of the inflammatory-immune central processes not being altered by peripheral inflammatory processes. To our knowledge, no CSF neopterin cut-off values to identify specific immune-mediated processes have been previously established. Moreover, data regarding the evaluation of CSF neopterin values in large cohorts of neurological patients have scarcely been reported [6], [21]. In our previous study [11] we found a statistically significant correlation between CSF neopterin and age in children younger than one month of age. Thus before starting the present study, we excluded all patients younger than one month of age. We established a CSF neopterin cut-off value of 61 nmol/L through the analysis and comparison of two cohorts of patients with and without confirmed CNS inflammatory-immune mediated processes. As it is known, during the early postnatal life there is a great variation in neuronal network formation, in blood-brain barrier stability and in amounts of neurotransmitters modulators [11], [22] leading to greater CSF neopterin values. We are concerned about the different mean age of both groups studied (6.5 years versus 8 months) and the possibilities of bias in our results due to those normal age-related changes in CSF neopterin. To test whether the difference in age had effect on the cut-off, we did two COR curves studies. One COR curve that included 1-month to 1-year-old patients (data not shown) and another one that contained 1-month to 19-years old patients (present COR curve). We obtained two cut-off values with very similar sensibility and specificity. That finding and the lack of correlation between CSF neopterin with the age in the 606 neurological patients corroborated the limited influence of the age in CSF neopterin values in our study. The newly established cut-off value was higher than the upper limit of most published reference intervals for neopterin, which are mainly clustered between 20–34 nmol/L [6], [10], [11], [12].

Interestingly, 56/606 neurological patients showed neopterin levels higher than 61 nmol/L. This observation was remarkable as most of these patients were not under suspicion of having an immune-mediated process (except for some of the 23 cases with inflammatory diseases). In contrast, if the former upper limit of CSF neopterin (34 nmol/L) would have been used, 87 out of 606 patients would have shown neopterin values above the normal range. Using this approach, the number of patients seems excessive because most of them belonged to clinical categories with no evidence of immune-mediated processes, such as neurometabolic diseases (data not shown). Nevertheless, only one case belonging to the inflammatory-immune mediated group presented a CSF neopterin value between 34 and 61 nmol/L. In all likelihood, with the application of this new cut-off value, a better discrimination between inflammatory-immune mediated conditions and other neurological conditions will be achieved. This discrimination may be critical for etiological diagnosis and therapeutic options because treatment strategies may be completely different in these categories.

Only a previous study about CSF neopterin values in neurological patients was published [6]. In that study, 158 cases were analyzed and grouped according to the mode of presentation and subsequent evolution of disease. We grouped our neurological patients according to whether they had a genetic, acquired or unknown etiologic neurologic disease or inflammatory-immune mediated process. This classification allowed us to identify which clinical groups had CSF neopterin values above the cut-off value. As expected, CSF neopterin was significantly elevated in the group of inflammatory-immune mediated processes [12]. However, 7 out of 23 patients in this group showed normal neopterin values, and the WBC and protein values were even less frequently altered (Tables 2 and 3), supporting the higher specificity and sensitivity of neopterin analysis compared with WBC and proteins, as previously suggested [6]. The relapsing course of these diseases and the normal cytokines and other immune biomarkers reported for patients with Aicardi-Goutieres syndrome [12] and some encephalitis [2], [6], [23] would explain these findings. Interestingly, patients from the other clinical groups also showed increased CSF neopterin values, with CSF protein and WBC being less frequently impaired than neopterin.

Concerning the neurological patients with genetic diseases, ten cases showed CSF neopterin levels moderately above 61 nmol/L (eight being patients with mitochondrial diseases). This subtle elevation most likely reflected inflammatory events associated with CNS damage [6] as these eight patients presented white matter disturbances (n = 2), brain atrophy (n = 1) and basal ganglia abnormalities (n = 5). The neurologic patients with other genetic diseases showed mostly normal neopterin concentrations given that no immune-mediated processes are present in these disorders, supporting the specificity of the analysis.

Regarding the patients with acquired neurologic disorders or with unknown etiology, although the percentage of patients with high CSF neopterin was quite similar to that of group A (7.1% versus 6.2%), some patients showed values in the range of inflammatory-immune-mediated diseases (Table 2). In these patients, the diagnostic orientation changed after neopterin analysis from a metabolic or a genetic disorder to an immune-mediated process.

White matter abnormalities on MRI were associated with CSF neopterin levels above 61 nmol/L in the group B patients. These results may be explained because neopterin is also produced intrathecally by different glial brain cells, acting as a possible link among inflammation, neurodegeneration and regeneration [24], [25]. These events can be found in primary inflammatory processes but are also commonly involved in some neurological acquired and unknown etiology diseases, in which neuroimaging reflects CNS damage. Stroke, epilepsy [16] and ischemia [20] are different processes that may generate hypoxia and production of reactive oxygen species leading to activation of inflammatory cascade. These inflammatory products and oxidative stress not only release proinflammatory cytokines but also alter the permeability of the blood-brain barrier, produce the disruption of neuronal-microgial interaction and increases in extracellular glutamate. All of these events may explain the high CSF neopterin values in patients belonging to Group B [20]. Furthermore, these patients would be candidates to receive a therapeutic assay with an anti-inflammatory treatment. In group B, no increased risk of having epilepsy was observed in the patients with neopterin >61 nmol/L. However, approximately 50% of the patients (table 2) with high neopterin presented with epilepsy. This matter deserves further investigation as a relationship between epilepsy and inflammation in the CNS has been previously reported [16] with a worse prognosis in patients presenting with epilepsy and increased neopterin [16].

Concerning patients with neopterin levels below 61 nmol/L (n = 550), nine patients showed increased WBC, and 66 showed increased proteins. Furthermore, in groups B and C, there was a clear difference between the percentage of patients with high CSF neopterin and those with high CSF WBC (figure 2), supporting the high sensitivity of the neopterin values. However, the CSF protein concentrations were frequently increased, most likely reflecting other pathophysiological phenomena in addition to immune events, such as brain injuries by vascular events [26] and increased blood-brain barrier permeability in energy metabolism disorders [27]. This finding would explain the relatively high percentage of increased CSF proteins compared with increased CSF WBC. Further evidence for neopterin as a good inflammatory biomarker was the association of its increased CSF values with a higher risk of having alterations in both WBC counts and protein concentration. This fact is important for the clinical practice since these two markers are the most commonly applied for diagnosis of inflammatory/immune events in clinical laboratories

## Conclusion

This report is the first to establish a new CSF neopterin cut-off value to detect inflammatory and immune-mediated neurological disorders. This new value was higher than the upper-limit of the reference intervals previously established. Although the group of patients who more frequently present high CSF neopterin values was that with immune-inflammatory processes, patients with other neurological disorders had CSF neopterin values above the new cut-off value in a noticeable percentage of cases. Therefore, this value was not only useful to detect the presence of an immune-mediated process but also because the possibility of an anti-inflammatory treatment may arise.
